# Molecular devolution in chronic myelomonocytic leukemia during treatment with decitabine/cedazuridine: A case report

**DOI:** 10.1007/s00277-026-06850-3

**Published:** 2026-02-04

**Authors:** Klaus Geissler, Gerlinde Mitterbauer-Hohendanner, Roland Jäger

**Affiliations:** 1 Medical School, Sigmund Freud Private University of Vienna, Vienna, Austria; 2 Karl Landsteiner Institute for Hematooncology Research, Vienna, Austria; 3 Department of Laboratory Medicine, Medical University of Vienna, Vienna, Austria

**Keywords:** Decitabine/cedazuridine, Chronic myelomonocytic leukemia, Disease modifying potential, Clonal hematopoiesis, Case report

## Abstract

Oral decitabine/cedazuridine has been studied in chronic myelomonocytic leukemia (CMML) recently but the molecular changes during this treatment have not been reported so far. We report a CMML patient who was treated with oral decitabine/cedazuridine and showed a stepwise loss of KRAS mutation and trisomy 8 while the TET2 mutated clone gradually increased eventually resulting in clonal hematopoiesis. This case report highlights the disease modifying potential of this treatment in single CMML patients.

## Introduction

Recently, Savona et al. published the efficacy and safety of oral decitabine/cedazuridine in the chronic myelomonocytic leukemia (CMML) subpopulations from phase 2 and 3 studies [1]. Although the prevalences and distributions of somatic mutations at diagnosis were shown serial determinations of molecular/genetic features during treatment were not reported in this paper. Here we demonstrate the changes of the mutational landscape in a CMML patient who was treated with decitabine/cedazuridine within the ASTX727-06 Extension Study.

 The stepwise accumulation of molecular aberrations during the process of progression of malignancy indicates evolution of disease and has been described in CMML by us and others [2–4]. The opposite phenomenon which we would like to call devolution implying a return to a less advanced stage or a decline in molecular complexity has not been described in CMML to our knowledge.

## Case

In 8/2022 we included a 66 years old female patient with CMML in the ASTX727-06 Extension Study who was treated with oral decitabine/cedazuridine (35 mg/100 mg) on days 1–5 every month. The study was approved by the ethics committee of the city of Vienna and the patient gave informed consent to the study.

The patient had elevated and rising monocyte counts starting in 7/2019 (1.4 G/L). Because of symptoms including weight loss, sweating and fatigue she was sent to our clinic in 6/2022. There were no hematologic malignancies in her family history, and in the medical history there were no comorbidities. In the physical examination we could not find peripheral lymphadenopathy, the spleen was palpabel 3 cm below left costal margin. In the sonography of spleen a length diameter of 15.3 cm was reported.

At diagnosis the blood picture showed a white blood cell count of 13.13 G/L, a monocyte count of 5.38 G/L, a hemoglobin value of 13.9 g/dL and a platelet count of 129 G/L. In the bone marrow (BM) 15% blasts were counted and the diagnosis CMML-2 was made according to the 2016 revision to the World Health Organization classification of myeloid neoplasms and acute leukemia [5]. At diagnosis molecular/genetic analyses showed KRAS mutation (c.38 > A) as determined by next generation sequencing (Myeloid Solution Genepanel [Sophia Genetics]) with a variant allele frequency (VAF) of 39%, 72% of cells with trisomy 8 as determined by fluorescence in situ hybridisation and a TET2 mutated clone (c.3955-2A > G) with a VAF of 4%.

After establishing diagnosis and giving informed consent the patient was screened for potential participation in the ASTX727-06 Extension Study since she was considered high-risk according to IPSS score [6]. Following inclusion she was treated with oral decitabine/cedazuridine (35 mg/100 mg) on days 1–5 every month. After 2 cycles of treatment there was a clear clinical improvement of symptoms, a drop of monocyte counts and a disappearance of palpable splenomegaly. In 2/2023 the patient achieved a complete response (CR) according to the response criteria proposed by the international consortium published by Savona et al. [7].

**Fig. 1:**
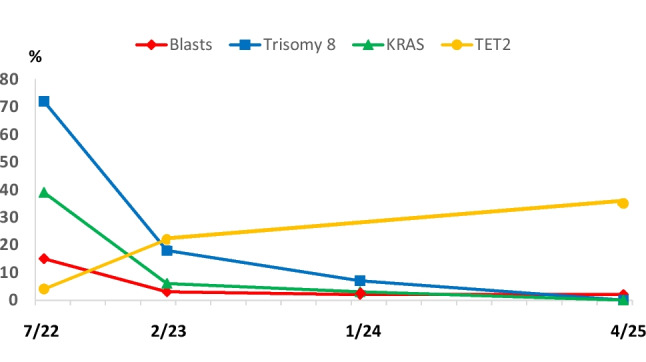
Changes of percentages of blast cells and molecular/genetic features in bone marrow during treatment with decitabine/cedazuridine

Serial molecular testing of BM was performed 6, 17 and 32 months following initiation of treatment. As shown in Fig. 1 there was a stepwise loss of molecular aberrations during treatment. In parallel with a decrease of blast cells from 15 to 3% 8 months later the percentages of trisomy 8 positive cells and of KRAS mutated cells markedly dropped during treatment with oral decitabine/cedazuridine and were not detectable at the last BM analysis in 4/2025. On the other hand the VAF of the TET2 mutated clone increased from 4% over time to 35% in 4/2025 with a completely normal blood count (WBC 8.67 G/L; Hb 14.9 g/dL; platelet count 260 G/L; monocyte count 0.7 G/L) indicating a return to TET2 mutated clonal hematopiesis [8]. Thus, evolution of CMML which commonly starts with a TET2 mutated cell clone was reversed during treatment [2–4].

## Discussion

A previous study with serial whole exome sequencing in CMML patients treated with hypomethylating agents including azacitidine and decitabine concluded that these agents do not alter the mutational allele burdens even in reponding patients [9]. Our case report shows that this conclusion cannot be applied to all patients and that there may be some patients in whom under oral decitabine/cedazuridine the complexity of the mutational landcape can be decreased and clonal hematopoiesis with normal blood picture restored. Thus, in these patients the evolution of disease which is a stepwise process, often starting with a TET2 mutated cell clone long time before manifestation of disease, cannot only be stopped but even reversed to its very early stage with no phenotypical features of disease which is a novel finding [2–4].

The major limitation of our observation is the fact that this is a case report in one patient and we were not able to do molecular analyses in more CMML patients treated with decitabine/cedazuridine. However, our data clearly indicate that some disease modifying activity of oral decitabine/cedazuridine can be observed at least in single CMML patients.

Disease modifying activity has been shown to be associated with survival benefit in other clonal myeloid diseases including chronic myeloid leukemia, primary myelofibrosis and polycythemia vera [10–12]. Thus, disease modifying activity of a drug seems to be a conditio sine qua non for treatment concepts aiming to achieve long lasting control of disease or even cure in some myeloid malignancies. The big challenge in future will be to more precisely identify CMML patients who are potential candidates for molecular devolution by decitabine/cedazuridine.

## Data availiblity

Data available on request due to privacy/ethical restrictions.
